# Comparative Pharmacological Effects of Tetracycline Derivatives on Osteoclast- and Osteoblast-Related Phenotypes and Skeletal Accumulation

**DOI:** 10.3390/ph19101610

**Published:** 2026-10-10

**Authors:** Saeka Shimochi, Katja Hrovat, Uruj Sarwar, Leire Bergara-Muguruza, Ari Alho, Maarit Laine, Akihisa Otaka, Yasuhiko Iwasaki, Ilkka Paatero, Ulvi K. Gursoy, Keijo Mäkelä, Eriika Savontaus, Jukka Salonen, Miho Nakamura

**Affiliations:** 1 Medicity Research Laboratory, Faculty of Medicine, University of Turku, Tykistökatu 6, 20520 Turku, Finland; saeka.shimochi@utu.fi (S.S.);; 2 Central Animal Laboratory, University of Turku, Itäinen Pitkäkatu 4 A, 20520 Turku, Finland; 3 Department of Anesthesia and Intensive Care, Turku University Hospital and University of Turku, Luolavuorentie 2, 20700 Turku, Finland; 4 Department of Chemistry and Materials Engineering, Faculty of Chemistry, Materials and Bioengineering, Kansai University, 3-3-35 Yamate-Cho, Suita-Shi, Osaka 5648680, Japan; 5 Turku Bioscience Centre, University of Turku and Åbo Akademi University, Tykistökatu 6, 20520 Turku, Finland; 6 Institute of Dentistry, University of Turku, Lemminkäisenkatu 2, 20520 Turku, Finland; 7 Department of Orthopaedics and Traumatology, Turku University Hospital and University of Turku, Luolavuorentie 2, 20700 Turku, Finland; keijo.makela@tyks.fi; 8 Institute of Biomedicine, University of Turku, Kiinamyllynkatu 10, 20520 Turku, Finland; 9 La Salle Campus Barcelona, Ramon Llull University, C/Sant Joan de la Salle 42, 08022 Barcelona, Spain; 10 Laboratory for Biomaterials and Bioengineering, Institute of Science Tokyo, 2-3-10 Kanda-Surugadai, Chiyoda, Tokyo 1010062, Japan

**Keywords:** bone remodeling, osteoporosis, osteoclastogenesis, extracellular matrix mineralization, tetracycline derivatives, drug repurposing

## Abstract

**Background/Objectives:** Tetracycline (TC) derivatives possess biological activities beyond their antimicrobial effects and exhibit affinity for mineralized tissues. However, direct comparisons of clinically established TC derivatives across bone-related experimental systems remain limited. This study comparatively evaluated TC, oxytetracycline (OC), doxycycline (DC), and minocycline (MC) to characterize their compound- and concentration-dependent effects on bone-related cellular phenotypes and skeletal accumulation. **Methods:** TC, OC, DC, and MC were evaluated using primary human osteoclasts, primary human mesenchymal stem cell (MSC)-derived osteoblasts, MG-63 osteoblast-like cells, and zebrafish embryos. Osteoclast formation and resorption, ALP-positive area, cellular metabolic activity, extracellular matrix mineralization, and compound localization were assessed. **Results:** All four derivatives were associated with concentration-dependent reductions in osteoclast formation, multinucleation, and resorptive activity, with DC showing pronounced effects. At 10 μg/mL, TC, OC, and MC reduced resorption pit volume to approximately 10–20% of untreated control values, while DC markedly reduced resorption even at 5 μg/mL. In primary MSC-derived osteoblasts, treatment with all four derivatives was associated with concentration-dependent reductions in ALP-positive area. In MG-63 cells, none reduced WST-8 metabolic activity at concentrations up to 20 μg/mL, whereas extracellular matrix mineralization showed compound- and concentration-dependent responses. All four derivatives preferentially localized to mineralized matrices in vitro and accumulated in developing skeletal structures in zebrafish, where increasing concentrations were associated with reduced mineralization intensity. **Conclusions:** Clinically established TC derivatives exhibit distinct compound- and concentration-dependent pharmacological profiles across bone-related experimental models and preferential affinity for mineralized tissues. These findings provide a comparative framework for future mechanistic studies of the bone-related biological effects and skeletal accumulation of TC derivatives.

## 1. Introduction

Osteoporosis develops when bone resorption by osteoclasts exceeds bone formation by osteoblasts, leading to a progressive decline in bone mass and greater susceptibility to fractures. Globally, osteoporosis represents a growing health concern, particularly in aging populations. Approximately one-third of women and one-fifth of men are estimated to sustain an osteoporotic fracture over the course of their lives [1]. Population aging is likely to further increase both the prevalence of osteoporosis and its associated socioeconomic burden, reinforcing the need for safe and effective therapeutic strategies.

Pharmacological management of osteoporosis primarily relies on agents that either suppress bone resorption or stimulate bone formation. The former includes bisphosphonates and inhibitors of receptor activator of nuclear factor κB ligand (RANKL), while the latter includes parathyroid hormone analogs and sclerostin-targeting monoclonal antibodies. Despite their established efficacy, these treatments have limitations. Long-term bisphosphonate use has been associated with atypical fractures, osteonecrosis of the jaw, and other adverse skeletal effects [2,3,4,5]. RANKL inhibition effectively increases bone mineral density but has also been associated with adverse effects, including osteonecrosis of the jaw [6]. In addition, the cost and long-term accessibility of some biologic therapies remain important considerations. These limitations continue to motivate the investigation of alternative pharmacological approaches to modulating bone remodeling.

Drug repurposing provides an opportunity to investigate clinically established compounds for biological activities beyond their original therapeutic indications. Tetracyclines (TCs), initially introduced as broad-spectrum antibiotics, are of particular interest because they exhibit several non-antimicrobial biological activities [7]. In addition to tetracycline (TC), clinically used derivatives include oxytetracycline (OC), doxycycline (DC), and minocycline (MC). DC has been reported to exert biological effects in diverse experimental contexts, including anticancer activity [8] and promotion of bone repair in vivo [9]. Tetracycline-related compounds have also been reported to influence osteoclast biology. For example, chemically modified TCs can induce osteoclast apoptosis in vitro [10], and TC analogues have been shown to suppress osteoclast differentiation through inhibition of matrix metalloproteinase (MMP)-9-mediated histone H3 cleavage [11]. Collectively, these observations indicate that TC derivatives can interact with cellular processes relevant to skeletal biology and provide a rationale for their further pharmacological characterization in bone-related experimental systems.

Importantly, individual TC derivatives differ in their physicochemical and pharmacokinetic properties [12]. TC and OC are less lipophilic and exhibit lower absorption than newer derivatives, whereas the semisynthetic compounds DC and MC are more lipophilic and are more completely absorbed. Structural modification of the upper peripheral region of the TC scaffold, particularly positions C7–C9 of the D ring, can alter biological activity [13,14]. The C4 dimethylamino group is required for antibacterial activity, whereas 4-demethylamino and other chemically modified TCs can retain or enhance non-antimicrobial biological activities [13,14,15]. These structural and pharmacological differences raise the possibility that individual TC derivatives may exert distinct effects on bone-related cellular phenotypes. However, direct comparative evaluation of clinically established TC derivatives across multiple bone-related experimental systems remains limited.

Bone remodeling involves the coupled actions of osteoclasts, which resorb bone, and osteoblasts, which form new bone [16], and is influenced by mechanical [17], metabolic [18], endocrine, and pharmacological factors [16]. Therefore, evaluation of candidate compounds in both osteoclast- and osteoblast-related systems is important for characterizing their potential effects on skeletal homeostasis. In addition, the well-established affinity of TCs for mineralized tissues provides a further rationale for examining their localization and effects within mineralized matrices. In the present study, we comparatively evaluated TC, OC, DC, and MC across human osteoclast cultures, primary human mesenchymal stem cell (MSC)-derived osteoblast cultures, MG-63 osteoblast-like cells, and a zebrafish model. We investigated their concentration-dependent effects on osteoclast formation and resorptive activity, osteoblast-related phenotypes and extracellular matrix (ECM) mineralization, as well as their localization within mineralized tissues. This comparative approach was designed to characterize compound- and concentration-dependent pharmacological profiles of clinically established TC derivatives and to provide an experimental basis for their further investigation in the context of skeletal drug repurposing.

## 2. Results

### 2.1. Effects of TC Derivatives on Osteoclastogenesis

**Figure 1:**
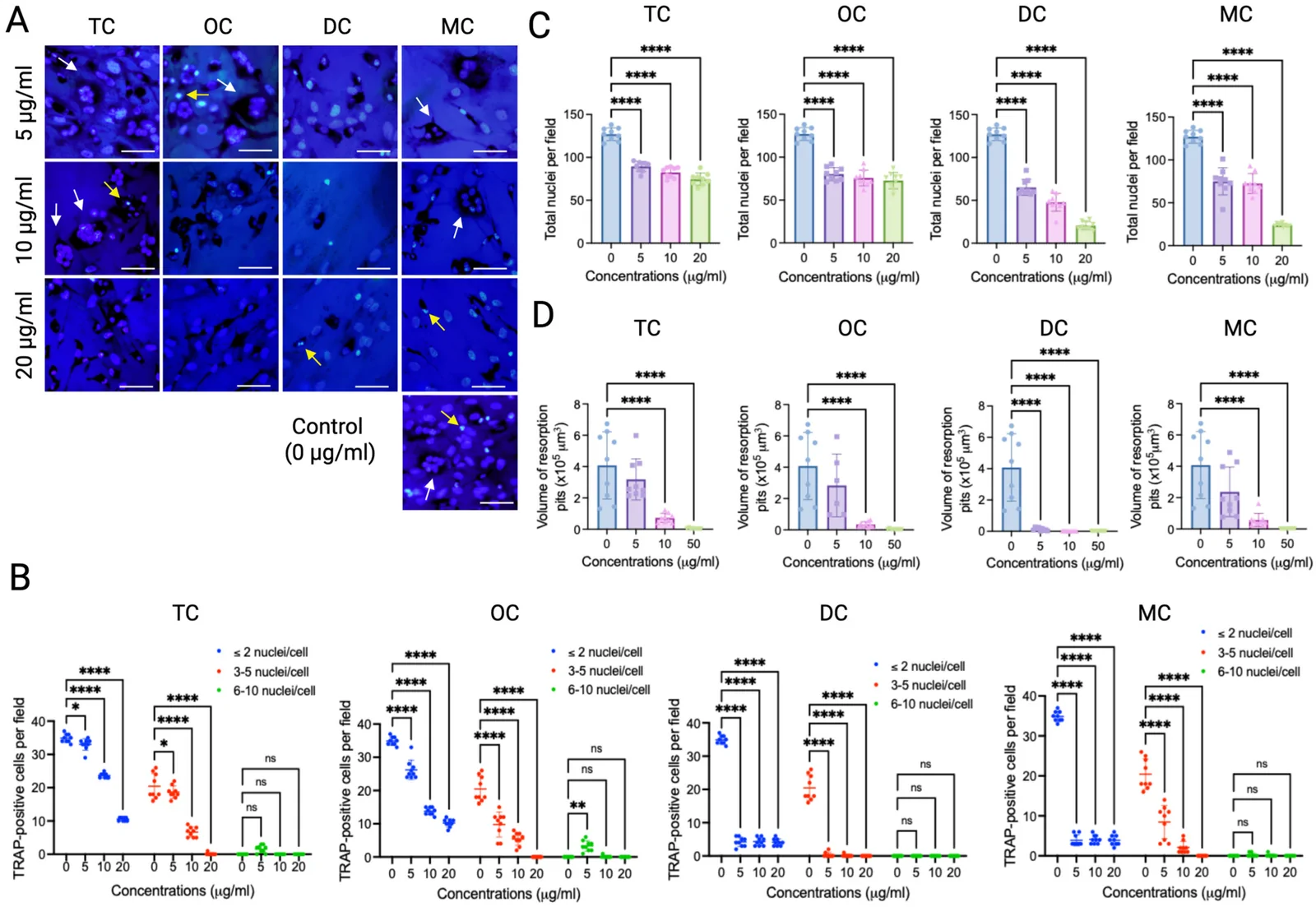
Effects of TC derivatives on osteoclast formation and bone resorptive activity. Human osteoclast precursors were cultured in the presence of TC, OC, DC, or MC at the indicated concentrations. (**A**) Representative fluorescence images of TRAP-stained osteoclast cultures following treatment with TC derivatives. White arrows indicate multinucleated TRAP-positive osteoclasts, whereas yellow arrows indicate nuclei exhibiting condensed or fragmented morphology. Scale bars = 50 μm. (**B**) Quantification of TRAP-positive cells according to the number of nuclei per cell (≤2, 3–5, and 6–10 nuclei). (**C**) Quantification of the total number of Hoechst-positive nuclei per microscopic field. (**D**) Quantification of resorption pit volume on bone slices. Treatment with TC derivatives was associated with concentration-dependent reductions in osteoclast multinucleation and bone resorptive activity. Data are presented as mean ± SD (n = 9). * *p* < 0.05, ** *p* < 0.01, **** *p* < 0.0001; ns, not significant, compared with the corresponding 0 μg/mL control group.

Human osteoclasts cultured on bone slices were exposed to different TC derivatives over a range of concentrations to assess osteoclast formation and resorptive activity. TRAP staining revealed that a variable number of bone marrow cells differentiated into osteoclasts (Figure 1A). There were TRAP-positive cells with a single nucleus, suggesting incomplete differentiation into mature osteoclasts. To further characterize osteoclast formation, TRAP-positive cells were classified according to the number of nuclei per cell. Increasing concentrations of each TC derivative were associated with progressively fewer multinucleated osteoclasts (Figure 1B). Cells containing 3–5 and 6–10 nuclei were markedly reduced at higher concentrations, while a similar trend was observed for cells containing > 10 nuclei (Supplementary Figure S1A). Quantification of total Hoechst-positive nuclei demonstrated a concentration-dependent reduction in the number of nuclei per microscopic field, particularly following DC and MC treatment (Figure 1C). To examine whether the reduction in osteoclast formation was accompanied by apoptotic nuclear morphology, nuclei exhibiting condensed or fragmented morphology were quantified. No statistically significant differences were detected among treatment groups (Supplementary Figure S1B).

Consistent with the reduction in multinucleated osteoclast formation, resorption pit volume was also significantly reduced following treatment with all TC derivatives (Figure 1D). Osteoclast resorption pits formed on bone slices were visualized by laser microscopy (Figure S1C). The largest resorption pits were observed in the untreated control group and in osteoclasts treated with low concentrations of TC (5 μg/mL) or OC (5 μg/mL). TC, OC, and MC were associated with concentration-dependent reductions in resorption pit volume, whereas DC markedly reduced resorption even at the lowest concentration tested (5 μg/mL). Compared with the untreated control, treatment with 10 μg/mL TC, OC, and MC reduced the resorption pit volume to approximately 10–20% of control values, while treatment with 20 μg/mL almost completely abolished detectable resorption. Similar trends were observed for resorption pit depth (Figure S1D). The marked reduction in resorption pit volume was consistent with the decrease in multinucleated osteoclasts observed following TC treatment.

To further characterize protease-related changes in osteoclast cultures, the levels of MMP-2 and MMP-9 in cell lysates were assessed after 14 days of osteoclast culture on bone slices in the presence of different TC derivatives (Supplementary Figure S2). MMP-2 levels were reduced in osteoclast cultures treated with higher concentrations of TC (10 and 20 μg/mL), OC (10 and 20 μg/mL), and MC (20 μg/mL). DC reduced MMP-2 levels at all tested concentrations (5, 10, and 20 μg/mL). MMP-9 levels were reduced in osteoclast cultures treated with all tested TC derivatives. Notably, all TC derivatives significantly reduced MMP-9 levels even at relatively low concentrations. These findings are presented as exploratory, supportive observations because the analyses were performed using whole-cell lysates and therefore do not identify the cellular source of MMP production or establish a causal relationship between MMP levels and osteoclast resorptive activity.

### 2.2. Effects of TC Derivatives on ALP Expression in Primary Human MSC-Derived Osteoblasts

**Figure 2:**
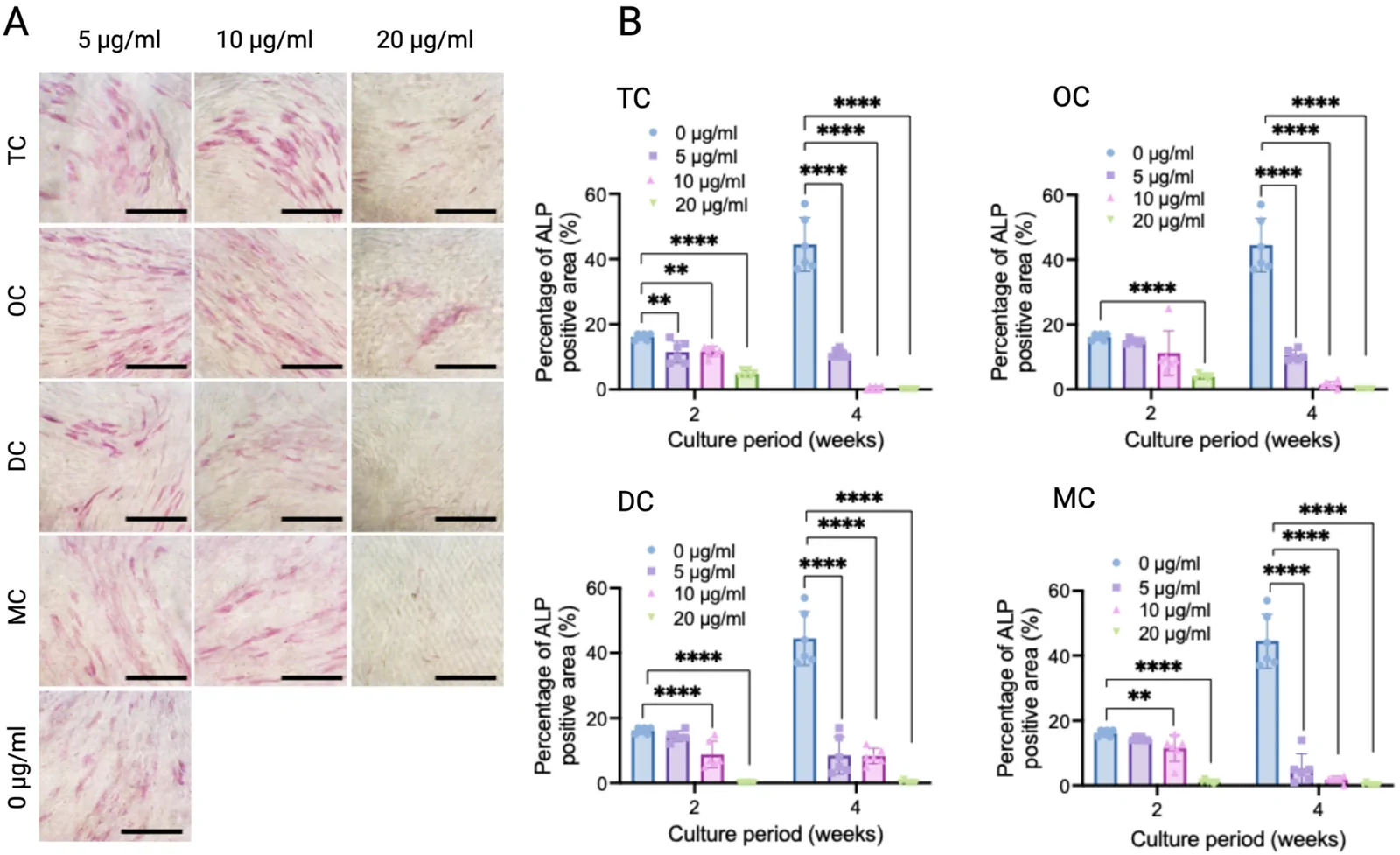
Effects of TC derivatives on ALP staining in primary human MSC-derived osteoblast cultures. Primary human MSC-derived osteoblasts were cultured in osteogenic medium containing TC, OC, DC, or MC at the indicated concentrations. ALP staining was evaluated after 2 and 4 weeks. (**A**) Representative ALP-stained images. Scale bar = 100 μm. (**B**) Quantitative analysis of the ALP-positive area. The percentage of ALP-positive area was determined by ImageJ image analysis. Treatment with all TC derivatives was associated with a concentration-dependent reduction in ALP-positive area, with more pronounced reductions after 4 weeks of culture. Data are presented as mean ± SD (n = 6). ** *p* < 0.01, **** *p* < 0.0001 compared with the corresponding 0 μg/mL control group.

Primary human MSC-derived osteoblasts were exposed to TC, OC, DC, or MC under osteogenic culture conditions, and ALP staining was assessed after 2 and 4 weeks (Figure 2). Representative ALP-stained images are shown in Figure 2A. All TC derivatives were associated with concentration-dependent reductions in ALP-positive area. Quantitative image analysis demonstrated a significant decrease in the percentage of ALP-positive area following treatment with TC, OC, DC, and MC (Figure 2B). The reduction was more pronounced after 4 weeks of culture, with little detectable ALP-positive staining at the highest concentration (20 μg/mL). Because cell viability was not directly assessed in these cultures, the contribution of reduced cell viability to the decrease in ALP-positive area cannot be determined. To further characterize the cellular responses to TC derivatives, MG-63 osteoblast-like cells were subsequently used to evaluate cellular metabolic activity, extracellular matrix mineralization, and compound localization.

### 2.3. Effects of TC Derivatives on Cellular Metabolic Activity and Mineralization of MG-63 Osteoblast-like Cells

**Figure 3:**
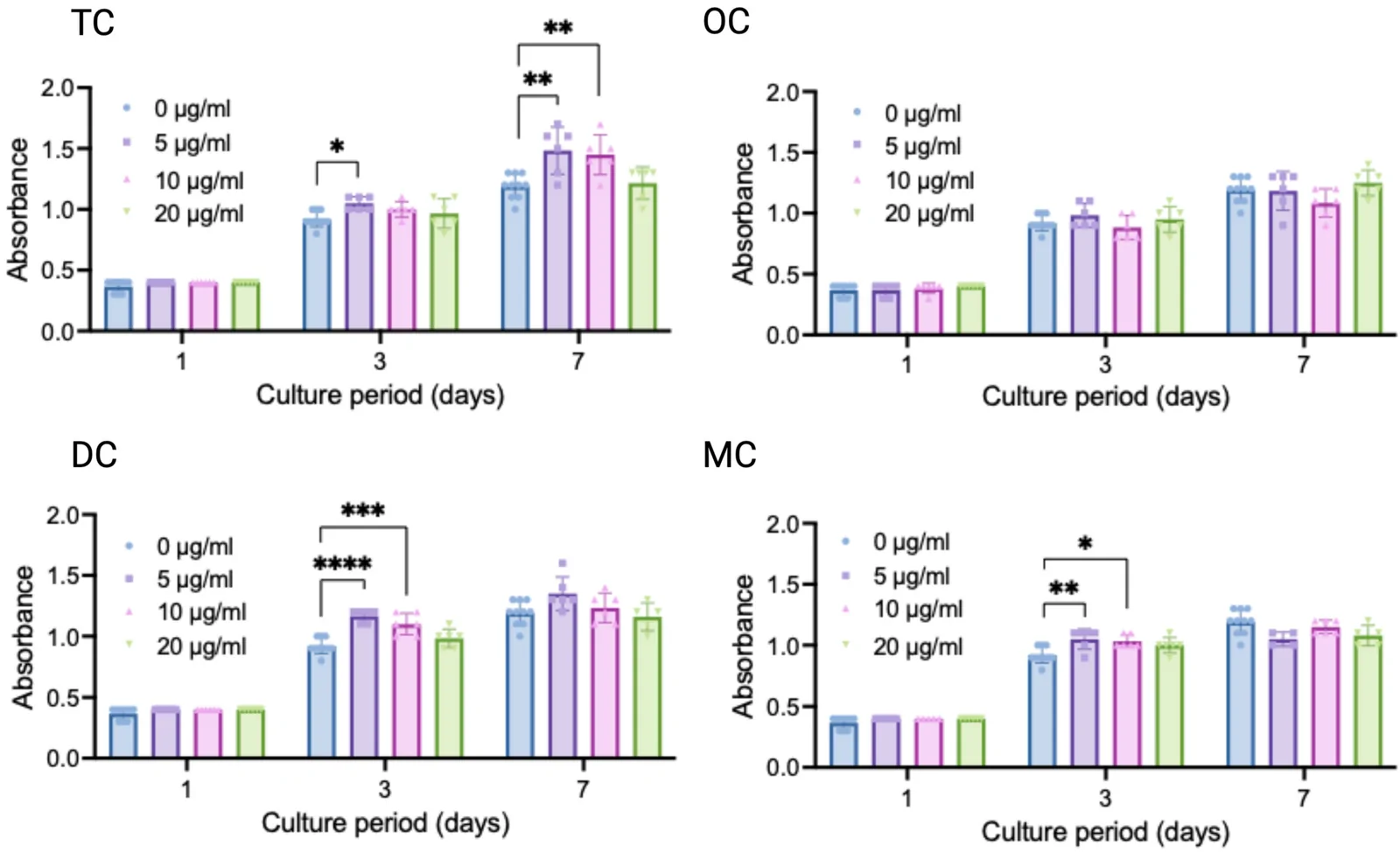
Effects of TC derivatives on cellular metabolic activity in MG-63 osteoblast-like cells. Cellular metabolic activity was assessed by WST-8 assay after 1, 3, and 7 days of culture in the presence of TC, OC, DC, or MC at the indicated concentrations. No significant differences were observed among groups at day 1. At day 3, WST-8 metabolic activity was significantly increased following treatment with low and moderate concentrations of TC, DC, and MC, whereas OC produced no significant changes. At day 7, increased WST-8 metabolic activity was maintained in TC-treated cultures at 5 and 10 μg/mL. None of the TC derivatives significantly reduced WST-8 metabolic activity relative to the untreated control at concentrations up to 20 μg/mL at any of the evaluated time points. Data are presented as mean ± SD (n = 10). * *p* < 0.05, ** *p* < 0.01, *** *p* < 0.001, **** *p* < 0.0001 compared with the corresponding 0 μg/mL control.

To evaluate the effects of TC derivatives on cellular metabolic activity, MG-63 osteoblast-like cells were cultured with TC, OC, DC, or MC at concentrations of 0, 5, 10, and 20 μg/mL, and WST-8 metabolic activity was assessed after 1, 3, and 7 days (Figure 3). At day 1, no significant differences were observed among the treatment groups. At day 3, WST-8 metabolic activity was significantly increased in cells treated with 5 μg/mL TC and with 5 and 10 μg/mL DC and MC compared with the untreated control, whereas OC had no significant effect. At day 7, significantly increased WST-8 metabolic activity was observed in cells treated with 5 and 10 μg/mL TC, while no significant differences were detected for OC, DC, or MC. Importantly, none of the four TC derivatives reduced WST-8 metabolic activity relative to the untreated control at concentrations up to 20 μg/mL at any of the evaluated time points.

**Figure 4:**
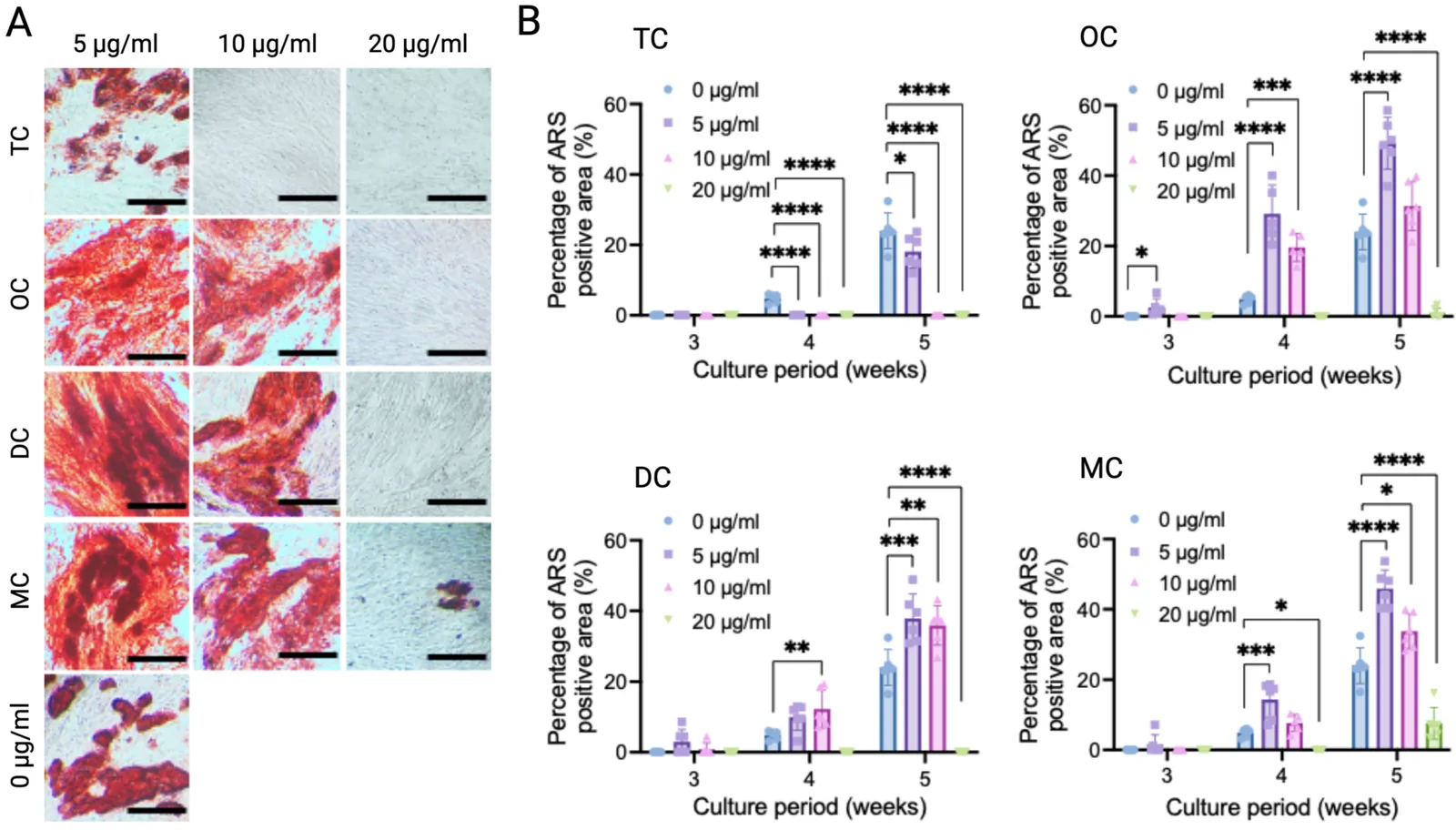
Effects of TC derivatives on mineralization during differentiation of MG-63 osteoblast-like cells. (**A**) Representative images of osteoblast cultures in osteoinductive medium showing the progression of mineralization in control cells. Initial mineral deposition was observed at 4 weeks, followed by extensive mineralized nodule formation at 5 weeks. Scale bar = 100 μm. (**B**) Mineralization of osteoblasts cultured with different TC derivatives at varying concentrations. TC and high concentrations (20 μg/mL) of OC, DC, and MC inhibited mineralized nodule formation, whereas low (5 μg/mL) and moderate (10 μg/mL) concentrations of OC, DC, and MC enhanced mineralization, indicating concentration-dependent changes in extracellular matrix mineralization. Data are presented as mean ± SD (n = 6). * *p* < 0.05, ** *p* < 0.01, *** *p* < 0.001, **** *p* < 0.0001 compared with the corresponding 0 μg/mL control.

To evaluate the effects of TC derivatives on extracellular matrix mineralization, MG-63 cells were cultured in osteoinductive medium with the same concentration range. Mineralization in untreated control cultures became detectable after 4 weeks and increased by 5 weeks (Figure 4A). TC reduced mineralized matrix formation, whereas OC, DC, and MC showed concentration-dependent biphasic responses: low and moderate concentrations (5 and 10 μg/mL) increased mineralized matrix formation, while the highest concentration (20 μg/mL) reduced mineralization (Figure 4B).

**Figure 5:**
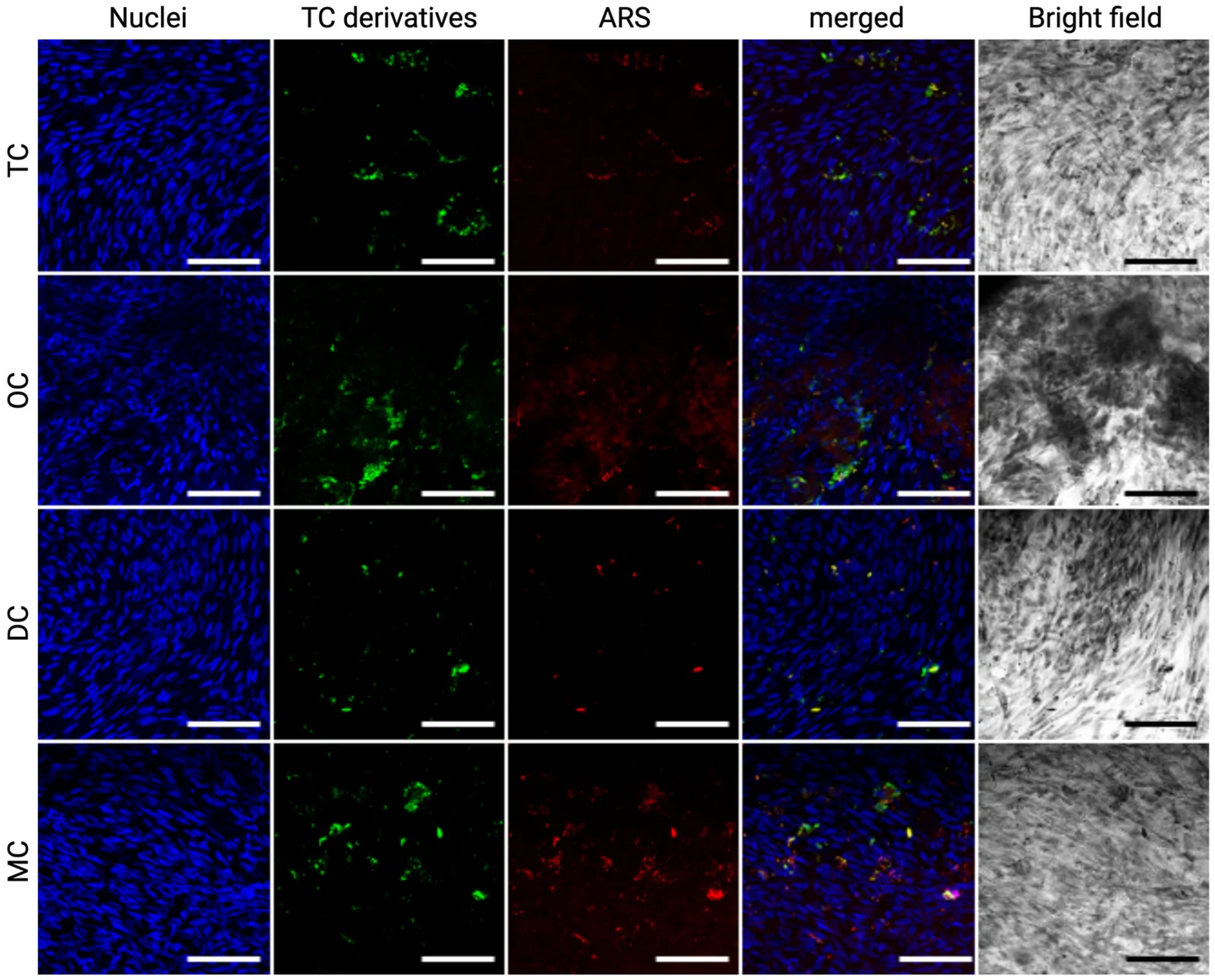
Localization of TC derivatives within mineralized bone nodules formed by osteoblasts. Representative confocal fluorescence microscopy images showing the distribution of TC, OC, DC, and MC in osteoblast cultures after 5 weeks of differentiation in osteoinductive medium. Cell nuclei were stained with Hoechst 33258 (blue), mineralized ECM was identified by ARS (red), and TC derivatives were visualized by their intrinsic fluorescence (green). Merged images demonstrate co-localization of TC derivatives with ARS-positive mineralized nodules, indicating preferential accumulation within mineralized bone matrix. Corresponding bright-field images show mineralized regions as needle-like structures. Scale bar = 100 μm.

The cellular localization of various TC derivatives in bone nodules produced by osteoblasts was examined using high-resolution confocal laser microscopy (Figure 5). Fluorescence imaging revealed an extensive distribution of nuclei, visualized in blue by Hoechst staining, across the entire surface of the glass coverslips. This uniform presence of nuclei indicated that the osteoblasts had reached confluence, thereby establishing a suitable environment for bone nodule formation and subsequent mineral deposition. The merged images demonstrated that all the tested TC derivatives were distributed in the mineralized regions of the bone nodules. These mineral deposits were identified by positive staining with ARS, confirming the presence of mineralized ECM. Furthermore, in the corresponding bright-field images acquired without a fluorescence filter, mineralized regions appeared as distinct, needle-like black structures, providing complementary morphological evidence of mineral deposition. The colocalization of TC derivatives with ARS-positive areas suggests that TC derivatives preferentially accumulate in mineralized bone matrix produced by osteoblasts.

### 2.4. Zebrafish as a Model of Skeletal Mineralization

**Figure 6:**
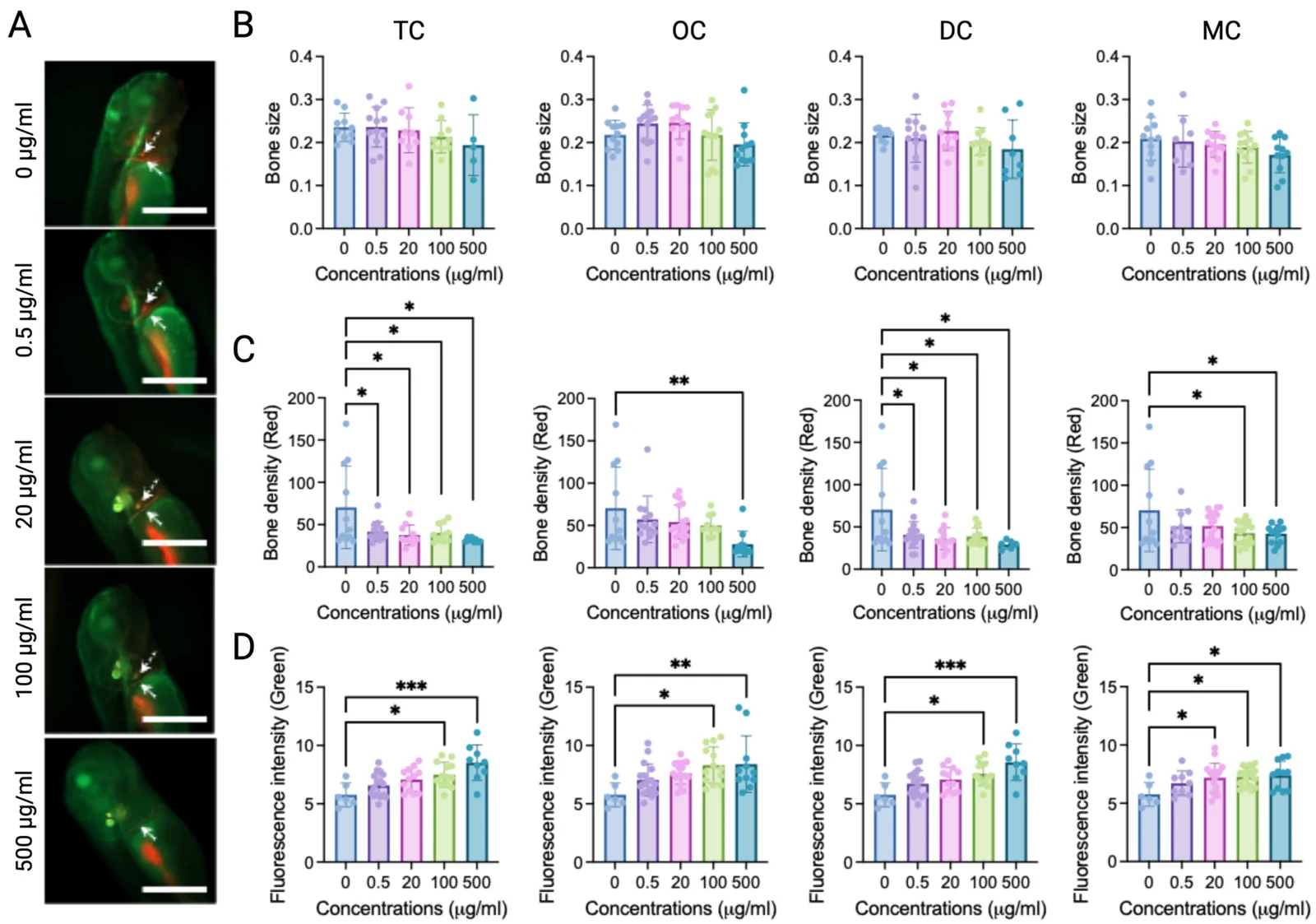
Effects of TC derivatives on craniofacial skeletal mineralization in zebrafish embryos. (**A**) Representative fluorescence images of zebrafish embryos at 4 days post-fertilization (dpf) showing the cleithrum and operculum following exposure to increasing concentrations of TC derivatives (TC shown as a representative example). Mineralized bone structures were visualized by fluorescence staining. The cleithrum is marked with arrows, and the operculum is marked with dotted-line arrows. Scale bar = 100 μm. (**B**) Quantification of cleithrum area in embryos treated with different concentrations of TC derivatives, showing no significant changes in bone size. (**C**) Quantitative analysis of mineralized bone density within the cleithrum region (red fluorescence channel), demonstrating a significant dose-dependent decrease with increasing TC derivative concentrations. (**D**) Quantification of TC derivative accumulation within the cleithrum region (green fluorescence channel), showing a significant dose-dependent increase in fluorescence intensity, indicating progressive compound localization in developing bone tissue. Data are presented as mean ± SD (n = 8–18). * *p* < 0.05, ** *p* < 0.01, *** *p* < 0.001 compared with the corresponding 0 μg/mL control.

To compare the effects of TC derivatives on skeletal mineralization, a zebrafish model was used to expose embryos to the different types of TC derivatives at several concentrations (Figure 6). Fluorescence imaging was performed to assess skeletal development, with a particular focus on the cleithrum and operculum regions, which are key indicators of early craniofacial bone formation. At 4 dpf, both bone structures were clearly developed and visualized under fluorescence microscopy in control embryos, confirming normal skeletal growth. In contrast, embryos treated with TC derivatives exhibited inhibited skeletal mineralization in a concentration-dependent manner (Figure 6A), with TC presented as a representative example.

Quantitative analysis revealed that the overall size of the cleithrum did not show significant differences across the tested concentrations of TC derivatives (Figure 6B). However, a marked decrease in cleithrum bone density was observed with increasing concentrations of the derivatives (Figure 6C), suggesting impaired mineralization during bone formation. Conversely, fluorescence intensity associated with TC derivatives within the cleithrum region increased significantly in a dose-dependent manner (Figure 6D), indicating progressive accumulation of these compounds in developing bone tissue. Collectively, these results demonstrate that TC derivatives preferentially localize to mineralized structures and may influence skeletal mineralization during zebrafish development in a concentration-dependent manner.

## 3. Discussion

The principal finding of this study is that four clinically established tetracycline derivatives exhibited distinct compound- and concentration-dependent pharmacological profiles across bone-related experimental systems. Although all four compounds were associated with reduced osteoclast formation and resorptive activity at higher concentrations, their effects on osteoblast-related parameters varied according to compound, concentration, and experimental model. Importantly, WST-8 metabolic activity in MG-63 cells was not reduced at concentrations up to 20 μg/mL, whereas extracellular matrix mineralization in these cells showed a biphasic response to several derivatives. In addition, all four compounds preferentially localized to mineralized matrices in vitro and to developing skeletal structures in zebrafish. Together, these observations provide a comparative pharmacological framework for further investigation of tetracycline derivatives in the context of skeletal drug repurposing.

Previous studies have examined the effects of individual TC derivatives, and comparative studies have evaluated selected osteogenic responses to multiple derivatives [19]. However, direct comparisons integrating osteoclast-related responses, osteoblast-related phenotypes, matrix mineralization, and skeletal accumulation remain limited. As summarized in Table S1, the present study extends previous observations by evaluating TC, OC, DC, and MC side-by-side across these complementary experimental systems. This integrated comparison revealed compound- and concentration-dependent profiles that would be difficult to identify from studies focusing on individual derivatives or selected biological endpoints.

### 3.1. Effects on Osteoclastogenesis

The present study demonstrated that treatment with TC and its derivatives was associated with concentration-dependent reductions in osteoclast formation, multinucleation, and bone resorptive activity in human osteoclast cultures. Among the compounds tested, DC consistently showed the most pronounced effects across all concentrations. These findings are consistent with previous studies demonstrating inhibitory effects of several TC derivatives on osteoclast formation and bone resorption [10,20]. TC and OC have been reported to inhibit osteoclast formation and resorptive activity in vitro and to affect bone turnover in vivo [21]. DC has also been reported to induce osteoclast apoptosis and inhibit osteoclast formation and resorption [22], while MC has been shown to inhibit osteoclast differentiation in association with suppression of MMP-9-mediated histone H3 cleavage [21].

To further characterize the changes observed in osteoclast cultures following treatment with TC derivatives, we analyzed the TRAP/Hoechst images. In addition to reducing the number of TRAP-positive multinucleated osteoclasts, TC derivatives decreased the total number of nuclei per microscopic field and reduced osteoclast multinucleation. However, no increase in condensed or fragmented nuclear morphology was observed, suggesting that the reduction in osteoclast formation was not accompanied by overt apoptotic nuclear changes under the present experimental conditions. Because dedicated apoptosis or viability assays were not performed, the present study cannot distinguish whether the reduced number of nuclei per microscopic field resulted from decreased precursor survival, altered proliferation, or impaired differentiation. The marked reduction in multinucleated osteoclasts may reflect effects on osteoclast differentiation and/or the cell–cell fusion process required for osteoclast maturation; however, because cell viability was not directly assessed, a contribution of reduced precursor survival cannot be excluded. Further studies using apoptosis- and viability-specific assays together with analyses of osteoclast fusion-related molecules will be required to clarify the underlying mechanisms.

To further characterize the biological effects of TC derivatives, we also evaluated MMP-2 and MMP-9 as exploratory markers of osteoclast-associated extracellular matrix remodeling. Following M-CSF- and RANKL-dependent differentiation from monocyte/macrophage precursors, osteoclasts degrade bone matrix through acidification and the release of proteolytic enzymes. MMPs participate in extracellular matrix turnover [23,24,25], with MMP-2 and MMP-9 being associated with several aspects of osteoclast biology, including migration and recruitment [23,26,27]. In the present study, MMP-2 and MMP-9 were evaluated as exploratory markers because previous studies have demonstrated that clinically established anti-resorptive agents, including bisphosphonates, suppress the expression and activity of multiple MMP family members [28]. We therefore investigated whether TC derivatives exhibit a similar biological profile. Because MMP measurements were performed using whole-cell lysates from mixed osteoclast cultures, the cellular source of MMP-2 and MMP-9 cannot be determined, and the observed changes cannot establish a causal relationship with osteoclast resorptive activity. These findings should therefore be interpreted as exploratory, supportive observations rather than mechanistic evidence of osteoclast-mediated bone resorption.

Among the tested compounds, DC consistently showed the most pronounced effects on osteoclast formation and resorption. This observation is consistent with previous reports describing relatively high lipophilicity and potent MMP-inhibitory activity of DC [29]. However, the present study did not directly investigate the mechanisms underlying the comparatively pronounced effects of DC, and no causal relationship between MMP modulation and the observed osteoclast responses can be established from these data. Future studies incorporating cathepsin K together with osteoclast differentiation and fusion markers will be required to clarify the molecular mechanisms underlying the observed changes in osteoclast formation and resorptive activity following treatment with TC derivatives. Collectively, these findings demonstrate concentration-dependent associations between TC derivative exposure and multiple osteoclast-related parameters and support further investigation of their biological effects in osteoporosis and other skeletal disorders. Previous studies have also suggested beneficial effects of TC derivatives in bone-related conditions, including rheumatoid arthritis [30] and aseptic loosening of prosthetic joint implants [31], providing additional context for investigating their biological effects in musculoskeletal diseases.

### 3.2. Effects on Osteoblast-Related Phenotypes and Extracellular Matrix Mineralization

In addition to their effects on osteoclasts, TC derivatives produced concentration-dependent changes in osteoblast-related phenotypes across the primary human MSC-derived osteoblast and MG-63 osteoblast-like cell models. In primary human MSC-derived osteoblast cultures, treatment with all TC derivatives was associated with concentration-dependent reductions in ALP-positive area. Because a dedicated viability assay was not performed in these primary cultures, the present data cannot distinguish a differentiation-specific effect from an effect related, at least in part, to reduced cell viability. Therefore, the reduction in ALP-positive area should not be interpreted as direct evidence of inhibition of osteogenic differentiation. To further characterize osteoblast-related cellular responses to TC derivatives, MG-63 osteoblast-like cells were used to evaluate cellular metabolic activity, extracellular matrix mineralization, and compound localization.

WST-8 analysis demonstrated that none of the four TC derivatives reduced cellular metabolic activity in MG-63 osteoblast-like cells at concentrations up to 20 μg/mL during the 7-day observation period. In contrast, low and moderate concentrations of TC, DC, and MC transiently increased WST-8 metabolic activity, whereas OC produced no significant changes. Because WST-8 reflects cellular metabolic activity rather than directly measuring cell number or proliferation, these findings should not be interpreted as evidence of enhanced proliferation. Previous studies have reported that substantially higher concentrations of TC can reduce osteoblast proliferation and metabolic activity, potentially through impairment of mitochondrial function [32]. These observations, together with the present findings, support the concept that cellular responses to TC derivatives are strongly concentration-dependent.

Interestingly, the effects on extracellular matrix mineralization differed from those observed in the WST-8 assay. Low and moderate concentrations of OC, DC, and MC increased mineralized matrix formation, whereas the highest concentration reduced mineralization. Because WST-8 metabolic activity was not reduced at these concentrations, the decrease in mineralized matrix formation at 20 μg/mL was not accompanied by a detectable reduction in cellular metabolic activity. These findings further indicate that the effects of TC derivatives on bone-related cellular phenotypes are both concentration- and compound-dependent.

The concentration- and compound-dependent effects on ECM mineralization observed in the present study are consistent with previous reports showing that TC derivatives can influence osteogenic differentiation and bone formation [19]. Previous studies have proposed several mechanisms by which TC derivatives may modulate osteogenic responses, including signaling pathways involved in osteoblast maturation and matrix production [19]. However, because these mechanisms were not investigated in the present study, the observed changes in mineralized matrix formation should be interpreted as phenotypic responses rather than evidence of a specific osteogenic mechanism.

Confocal microscopy demonstrated accumulation of TC derivatives within mineralized bone nodules produced by osteoblasts. This observation is consistent with the well-established affinity of TCs for mineralized tissues and supports previous reports describing their use as bone-labeling agents [22]. The ability of these compounds to localize within mineralized matrix may contribute to their prolonged retention within mineralized tissues and may partially explain their sustained effects on bone cells. Further studies using primary human osteoblasts and in vivo mammalian models are warranted to confirm these findings and determine their relevance to osteoporosis and other metabolic bone disorders.

### 3.3. Zebrafish as a Model for Skeletal Mineralization

The zebrafish model provided complementary in vivo evidence of the skeletal accumulation of TC derivatives and their association with changes in mineralization during development. Although no significant changes in cleithrum size were observed, increasing concentrations of TC derivatives were associated with reduced mineralization intensity and increased compound-derived fluorescence within mineralized skeletal structures, indicating concentration-dependent skeletal accumulation.

The affinity of TC derivatives for mineralized tissues has long been recognized and forms the basis for their widespread use as fluorescent markers of bone formation and mineralization [22]. The present findings extend these observations by comparatively demonstrating skeletal localization of TC, OC, DC, and MC and showing that their accumulation was accompanied by concentration-dependent changes in mineralization. These in vivo observations complement the in vitro findings in MG-63 osteoblast-like cells, in which TC derivatives localized to mineralized extracellular matrix and produced compound- and concentration-dependent changes in matrix mineralization.

An important aspect of the present study is the consistent localization of all four TC derivatives to mineralized structures in both the cell culture and zebrafish models. This finding is consistent with the established affinity of TCs for mineralized tissues and provides an additional pharmacological context for interpreting their effects in bone-related experimental systems. However, the biological consequences of skeletal accumulation and its relationship to internal drug exposure require further investigation.

It should be noted that the zebrafish model was used primarily to evaluate skeletal accumulation and developmental responses to TC derivatives [33,34]. The concentration range used in the zebrafish experiments was selected to compare the skeletal accumulation and developmental responses of the four TC derivatives over a broad range of exposure rather than to reproduce clinically achievable plasma concentrations [35,36]. Importantly, the nominal concentrations in the exposure medium do not directly represent the concentrations within the embryos. TC derivatives are relatively hydrophilic compounds with limited passive absorption, and based on published relationships between physicochemical properties and internal exposure in zebrafish embryos [37], intraembryonic exposure is expected to represent only a fraction of the nominal medium concentration. Nevertheless, internal drug concentrations were not directly measured in the present study, and systemic or developmental toxicity at the higher exposure concentrations cannot be excluded. Consequently, the reduced mineralization observed at higher concentrations may reflect developmental effects and should not be interpreted as direct evidence of altered bone remodeling or therapeutic efficacy in osteoporosis. Rather, the zebrafish findings provide complementary in vivo evidence that TC derivatives accumulate within developing mineralized tissues and are associated with concentration-dependent changes in skeletal mineralization. Further studies incorporating direct measurement of internal drug exposure and appropriate mammalian models will be required to determine the pharmacological and biological relevance of these observations to bone remodeling.

Although the four TC derivatives exhibited distinct profiles across the osteoclast- and osteoblast-related experimental systems, these differences cannot be directly attributed to their skeletal accumulation. All four derivatives showed preferential localization to mineralized matrices and developing skeletal structures; however, the present study did not quantitatively correlate skeletal accumulation with the magnitude of the cellular responses. Moreover, osteoclast- and osteoblast-related responses were evaluated in separate experimental systems, and the balance between bone resorption and bone formation was not directly assessed. Thus, skeletal affinity and cellular pharmacological activity should be considered complementary but distinct properties in the present study. Skeletal accumulation may influence local exposure of bone-associated cells, but whether compound-specific accumulation contributes to the balance between bone resorption and bone formation will require further investigation using quantitative skeletal pharmacokinetics, coupled osteoclast–osteoblast models, and appropriate in vivo bone-remodeling models.

### 3.4. Study Limitations

Several limitations of this study should be acknowledged. First, dedicated quantitative viability assays were not performed in either the primary human osteoclast cultures or the primary human MSC-derived osteoblast cultures. Although quantitative analysis of Hoechst-stained osteoclast cultures provided information on total nuclear number and nuclear morphology, these measurements do not constitute a direct assessment of cell viability. Therefore, reduced cell viability cannot be excluded as a contributing factor to the observed reductions in osteoclast formation, multinucleation, and resorptive activity. Similarly, the concentration-dependent reduction in ALP-positive area observed in primary human MSC-derived osteoblast cultures cannot be attributed specifically to altered osteogenic differentiation because viability was not independently assessed. Accordingly, these findings should be interpreted as treatment-associated phenotypic changes rather than differentiation-specific effects.

Second, the osteoclast precursor population was not further characterized (e.g., by CD14/CD11b expression), and therefore potential differences in precursor composition could not be assessed. Third, osteoblast-related responses were evaluated using both primary human MSC-derived osteoblasts and the MG-63 osteoblast-like cell line. Detailed analyses of cellular metabolic activity, extracellular matrix mineralization, and compound localization were performed using MG-63 cells because of the limited availability of primary human bone marrow samples during the COVID-19 pandemic. Although WST-8 analysis provided information on cellular metabolic activity in MG-63 cells, these findings cannot be extrapolated to the primary human cell cultures. Further studies using primary human cells with dedicated viability assays will therefore be required to distinguish effects on cell survival from those on differentiation and other cellular responses. Fourth, MMP-2 and MMP-9 were evaluated only as exploratory markers, and their cellular sources and mechanistic roles were not investigated. Finally, the zebrafish model was used primarily to evaluate skeletal accumulation and developmental responses to TC derivatives and does not reproduce bone remodeling in adult mammals. Further studies in appropriate mammalian models will therefore be required to determine the relevance of these findings to adult skeletal homeostasis and disease.

## 4. Materials and Methods

### 4.1. Osteoclast Culture

Human bone marrow was used as the source of osteoclast precursors under a donation protocol approved by the Ethics Committee of Turku University Hospital (ETMK Dnro: 42/1801/2019). Bone marrow samples were collected from the femoral collum and trochanteric regions of male and female patients aged 49–70 years during hip replacement surgery, following a previously described procedure [38]. The samples were initially maintained in 75-cm^2^ tissue culture flasks for 1 day, after which the nonadherent cell fraction was collected and the flasks were rinsed with PBS. Mononuclear cells were then enriched by Ficoll-Paque (GE Healthcare, Chicago, IL, USA) density-gradient centrifugation, washed twice with PBS, and resuspended in α-minimum essential medium (α-MEM; Sigma–Aldrich, St. Louis, MO, USA) containing 10% fetal bovine serum (FBS; Gibco, Waltham, MA, USA), L-glutamine (Gibco, Waltham, MA, USA), and 100 U/mL penicillin–streptomycin (Gibco, Waltham, MA, USA).

The enriched mononuclear cell fraction was plated on bovine bone slices at 1 × 10^6^ cells/cm^2^. Cultures were exposed to TC, OC, DC, or MC at 0, 5, 10, or 20 μg/mL; all compounds were dissolved in sterile distilled water. TC and OC were obtained from Sigma–Aldrich (St. Louis, MO, USA), and DC and MC from Sigma–Aldrich (St. Louis, MO, USA). Osteoclast differentiation was induced with 50 ng/mL RANKL (PeproTech, Cranbury, NJ, USA), 25 ng/mL macrophage colony-stimulating factor (M-CSF; R&D Systems, Minneapolis, MN, USA), 5 ng/mL transforming growth factor beta (TGF-β; R&D Systems, Minneapolis, MN, USA), and 1 mM dexamethasone (Sigma–Aldrich, St. Louis, MO, USA), as previously described [39,40]. Cultures were maintained for 14 days at 37 °C in a humidified atmosphere containing 5% CO_2_, with half of the culture medium replaced every 3–4 days.

### 4.2. Osteoclast Differentiation and Resorption

After 14 days of culture on bovine bone slices, cells were fixed with 4% paraformaldehyde for 20 min. Osteoclast differentiation was evaluated by tartrate-resistant acid phosphatase (TRAP; Sigma–Aldrich, St. Louis, MO, USA) staining together with Hoechst 33258 (Sigma–Aldrich, St. Louis, MO, USA) nuclear staining. TRAP-positive multinucleated cells were counted per unit area on each bone slice. A total of at least 6 fields on 6 bone slices were counted to obtain an average for each experiment.

To further characterize osteoclast formation, the original TRAP/Hoechst fluorescence images were subjected to additional quantitative analysis with ImageJ software (version 1.54f; National Institutes of Health, Bethesda, MD, USA). Hoechst-positive nuclei were quantified to determine the total number of nuclei per microscopic field. TRAP-positive cells were classified according to the number of nuclei per cell as ≤2, 3–5, 6–10, or >10 nuclei [41,42]. In addition, nuclei exhibiting condensed or fragmented morphology were manually identified and expressed as a percentage of the total number of Hoechst-positive nuclei. Quantification of total nuclei was performed using the particle analysis function in ImageJ after applying a consistent threshold to all images.

After fluorescence microscopy, cells were gently removed from each bone slice by scrubbing, followed by rinsing with distilled water. Osteoclastic resorption was evaluated from the morphology of the resulting resorption pits. After drying, the bone slices were imaged using a color laser microscope equipped with an image analysis system (OLS4100; Olympus, Tokyo, Japan). Resorption pit volume was quantified from one field located in the central region of each bone slice. Resorption pit depth was determined from five measurements obtained within the analyzed field of each bone slice, and the mean value for each bone slice was used for statistical analysis.

### 4.3. Osteogenic Differentiation of Primary Human MSCs

Bone marrow collected from patients undergoing hip replacement surgery was used to obtain primary human mesenchymal stem cells (MSCs), following approval by the Human Subjects Committee of the University of Turku and a previously described protocol. Cells from the bone marrow aspirates were plated in tissue culture flasks, and the adherent MSC population was expanded in α-MEM containing 10% FBS, 2 mM L-glutamine, 100 U/mL penicillin–streptomycin, and 24 mM HEPES. At approximately 70% confluence, cells were released with trypsin–EDTA and transferred to 96-well plates at 5 × 10^4^ cells/cm^2^. For osteogenic induction, the culture medium contained 100 nM dexamethasone (Sigma–Aldrich, St. Louis, MO, USA), 10 mM β-glycerophosphate (Sigma–Aldrich, St. Louis, MO, USA), and 50 μg/mL ascorbic acid (Sigma–Aldrich, St. Louis, MO, USA). TC, OC, DC, or MC was added at 0, 5, 10, or 20 μg/mL. Cells were maintained at 37 °C under humidified 5% CO_2_ conditions, with half of the culture medium renewed every 3–4 days. ALP staining was performed after 2 and 4 weeks of osteogenic culture.

### 4.4. ALP Staining and Quantitative Image Analysis

ALP staining was performed with the Leukocyte Alkaline Phosphatase Kit (Sigma-Aldrich, St. Louis, MO, USA) following the protocol provided by the manufacturer. Representative images were acquired using a light microscope under identical imaging conditions. The percentage of ALP-positive area was quantified using ImageJ software (version 1.54f; National Institutes of Health, Bethesda, MD, USA) by applying a constant threshold to all images and expressing the positively stained area as a percentage of the total image area.

### 4.5. Cellular Metabolic Activity of MG-63 Osteoblast-like Cells Assessed by WST-8 Assay

MG-63 human osteoblast-like cells were cultured in Dulbecco’s modified Eagle medium (DMEM; Gibco, Waltham, MA, USA) containing 10% FBS, 100 U/mL penicillin–streptomycin, and 2 mM L-glutamine. Cultures were maintained at 37 °C under humidified conditions with 5% CO_2_. At approximately 70% confluence, the cells were detached with 0.05% trypsin–EDTA (Gibco, Waltham, MA, USA). The cell suspension was used for subculture or further experiments. The medium was changed every 3–4 days.

Cells were seeded into 96-well cell culture plates at a density of 5 × 10^3^ cells/cm^2^ and cultured with 0, 5, 10 and 20 µg/mL TC, OC, DC, and MC. After 1, 3, and 7 days, cellular metabolic activity was assessed using a WST-8 assay kit (Dojindo Molecular Technologies, Rockville, MD, USA; CK04) according to the manufacturer’s instructions.

### 4.6. ECM Mineralization of MG-63 Osteoblast-like Cells

MG-63 cells were plated in 96-well culture plates at 1 × 10^4^ cells/cm^2^ and allowed to reach confluence. Osteogenic induction was then initiated in medium containing 100 nM dexamethasone, 10 mM β-glycerophosphate, and 50 μg/mL ascorbic acid, with TC, OC, DC, or MC added at 0, 5, 10, or 20 μg/mL. Cultures were maintained at 37 °C under humidified conditions with 5% CO_2_. At 3, 4, and 5 weeks, cells were fixed with 4% paraformaldehyde for 20 min and stained with 40 mM alizarin red S (ARS; Sigma–Aldrich, St. Louis, MO, USA) to visualize extracellular matrix mineralization. Stained cultures were observed under a fluorescence microscope (Olympus IX71, Tokyo, Japan). Whole images of each 96-well plate were captured to obtain the average percentage of ARS-positive areas and to compare the effects of the TC derivatives on ECM mineralization.

### 4.7. Localization of TC Derivatives in Bone Nodules

MG-63 cells were plated at 5 × 10^3^ cells/cm^2^ on glass coverslips placed in 48-well culture plates and allowed to reach confluence. Osteogenic induction was subsequently initiated using medium containing 100 nM dexamethasone, 10 mM β-glycerophosphate, and 50 μg/mL ascorbic acid, together with 10 μg/mL TC, OC, DC, or MC. Cultures were maintained for 5 weeks at 37 °C under humidified conditions with 5% CO_2_ and then fixed with 4% paraformaldehyde for 20 min. For visualization of TC derivative localization within osteoblast-derived mineral deposits, cells were stained with Hoechst 33258 and 40 mM ARS, mounted in glycerol, and examined using a high-resolution confocal laser microscope (Zeiss LSM880; Carl Zeiss Microscopy GmbH, Jena, Germany) [43].

### 4.8. Zebrafish Model

Wild-type (WT) zebrafish embryos were maintained at the Zebrafish Core Facility at Turku Bioscience. Several male and female adult zebrafish (1:1 ratio) were chosen and placed in individual breeding chambers for 24 h. At 0 days postfertilization (dpf), the adult fish were transferred back to home tank. The embryos were collected using a strainer and placed into a Petri dish with E3 medium containing 5 mM NaCl, 0.17 mM KCl, 0.33 mM CaCl_2_·2H_2_O, 0.33 mM MgCl_2_·6H_2_O (pH 7.2–7.3), and 0.57% methylene blue (Sigma–Aldrich, St. Louis, MO, USA). The fertilized embryos were identified using a light microscope. The fertilized embryos were transferred into separate Petri dishes with E3 medium and 0.2 mM 1-phenyl 2-thiourea (PTU; Sigma–Aldrich, St. Louis, MO, USA) to block melanin synthesis and render the embryos optically transparent. At 1 dpf, 2 mg/mL pronase (Sigma–Aldrich, St. Louis, MO, USA) was added to the Petri dish to facilitate hatching. The Petri dish was kept in an incubator (+28.5 °C). The 2-dpf zebrafish embryos were exposed to 0, 0.5, 20, 100, and 500 µg/mL TC, OC, DC, and MC in E3 medium supplemented with 0.2 mM PTU for 2 days.

At 4 dpf, the zebrafish embryos were stained with 0.01% ARS (Sigma–Aldrich, St. Louis, MO, USA) diluted in E3 medium (pH 7.4) [44] at 28.5 °C for 15 min. After the zebrafish embryos were washed twice with E3 medium, the embryos were kept in E3 medium supplemented with 0.2 mM PTU and 200 mg/L tricaine for anesthesia. The embryos were observed under a fluorescence microscope (Zeiss AxioZoom V16 Stereomicroscope; Carl Zeiss Microscopy GmbH, Jena, Germany). EGFP (green channel, excitation filter BP 470/40 nm, emission filter BP 525/50 nm) was used to visualize the TC derivatives, and Texas Red (red channel, excitation filter BP 560/40 nm, emission filter BP 630/75 nm) and a Hamamatsu sCMOS Orca Flash4.0 LT + camera (Hamamatsu Photonics K.K., Hamamatsu, Japan) were used to visualize the ARS staining. The microscope was operated with ZEISS ZEN software (version 3.7), which was further used for image processing of the raw image data. To quantitatively assess the effects of TC derivatives on zebrafish skeletal mineralization, the cleithrum area as well as the fluorescence intensities corresponding to mineralized bone (red channel) and TC derivatives (green channel) were measured for each image using ImageJ software (version 1.54f).

### 4.9. Statistical Analysis

Data are presented as mean ± standard deviation (SD). Prior to statistical analysis, data were assessed for normality using the normality test function implemented in GraphPad Prism (version 11; GraphPad Software, San Diego, CA, USA). Homogeneity of variance was also confirmed before performing parametric statistical analysis. Comparisons among groups were conducted using one-way analysis of variance (ANOVA) followed by Tukey’s multiple-comparison test. Statistical significance was defined as *p* < 0.05.

## 5. Conclusions

In conclusion, the four clinically established TC derivatives examined in this study exhibited distinct compound- and concentration-dependent effects across bone-related experimental systems. In primary human osteoclast cultures, TC derivatives were associated with reductions in osteoclast formation, multinucleation, and resorptive activity, whereas treatment of primary human MSC-derived osteoblast cultures was associated with concentration-dependent reductions in ALP-positive area. In MG-63 osteoblast-like cells, TC derivatives produced compound- and concentration-dependent changes in cellular metabolic activity and ECM mineralization, without reducing WST-8 metabolic activity at concentrations up to 20 μg/mL. In addition, all four derivatives preferentially localized to mineralized matrices in vitro and developing skeletal structures in zebrafish. Collectively, these findings demonstrate distinct pharmacological profiles among TC, OC, DC, and MC across bone-related experimental models and provide a comparative framework for future mechanistic studies of their bone-related biological effects and skeletal accumulation.

## Data Availability

The original contributions presented in this study are included in the article/Supplementary Material. Further inquiries can be directed to the corresponding author.

## Supplementary Materials

The following supporting information can be downloaded at: https://www.mdpi.com/article/10.3390/ph19101610/s1. Supplementary Methods: Measurement of MMP-2 and MMP-9 levels in osteoclast cultures by ELISA; Figure S1: Quantitative analysis of osteoclast morphology and resorption following treatment with TC derivatives; Figure S2: Effects of TC derivatives on MMP-2 and MMP-9 levels in osteoclast cultures. Table S1: Comparison of previously reported bone-related effects of TC derivatives with the findings of the present study.
