# Supplementary material for: Comparative Pharmacological Effects of Tetracycline Derivatives on Osteoclast- and Osteoblast-Related Phenotypes and Skeletal Accumulation
Source: Pharmaceuticals (Basel). 2026 Oct 10;19(10):1610. doi: 10.3390/ph19101610 (PMC13708721; doi:10.3390/ph19101610)
Supplement: Supplementary file 1 [file pharmaceuticals-19-01610-s001.zip › pharmaceuticals-4597785-supplementary.pdf]

Supplementary Information for

Comparative Pharmacological Effects of Tetracycline Derivatives on Osteoclast- and Osteoblast-Related Phenotypes and Skeletal Accumulation

Saeka Shimochi[1,2], *Katja Hrovat*[1], *Uruj Sarwar*[1], *Leire Bergara-Muguruza*[1], *Ari Alho*[3], *Maarit Laine*[3], *Akihisa Otaka*[4], *Yasuhiko Iwasaki*[4], *Ilkka Paatero*[5], *Ulvi K. Gursoy*[6], *Keijo Mäkelä*[7], *Eriika Savontaus*[8], *Jukka Salonen*[1], *Miho Nakamura*[1, 9, 10]*

[1] Medicity Research Laboratory, Faculty of Medicine, University of Turku, Tykistökatu 6, 20520, Turku, Finland

[2] Central Animal Laboratory, University of Turku, Itäinen pitkäkatu 4 A, 20520 Turku, Finland

[3] Department of Anesthesia and Intensive Care, Turku University Hospital and University of Turku, Luolavuorentie 2, 20700 Turku, Finland

[4] Department of Chemistry and Materials Engineering, Faculty of Chemistry, Materials and Bioengineering, Kansai University, 3-3-35 Yamate-cho, Suita-shi, Osaka, 5648680, Japan

[5] Turku Bioscience Centre, University of Turku and Åbo Akademi University, Tykistökatu 6, 20520, Turku, Finland

[6] Institute of Dentistry, University of Turku, Lemminkäisenkatu 2, 20520 Turku, Finland

[7] Department of Orthopaedics and Traumatology, Turku University Hospital and University of Turku, Luolavuorentie 2, 20700 Turku, Finland

[8] Institute of Biomedicine, University of Turku, Kiinamyllynkatu 10, 20520 Turku, Finland

[9] La Salle Campus Barcelona Ramon Llull University, C/ Sant Joan de la Salle 42, 08022 Barcelona, Spain

[10] Laboratory for Biomaterials and Bioengineering, Institute of Science Tokyo, 2-3-10 Kanda-Surugadai, Chiyoda, Tokyo 1010062 Japan

Supplementary Materials

Measurement of MMP-2 and MMP-9 levels in osteoclast cultures by ELISA

Osteoclast precursors were cultured on bone slices with 0, 5, 10 and 20 µg/ml TC, OC, DC, and MC. After 14 days, the cells were lysed with 200 µl of lysis buffer containing 50 mM Tris-HCl (pH 6), 150 mM NaCl and 1% Triton X-100. The cells were scraped, collected into tubes, and sonicated for 10 seconds. The cell lysates were frozen at −20 °C until use. The concentrations of MMP−9 and MMP−2 were detected with Luminex technique using commercial multiplex kits (Milliplex® Human MMP Magnetic Bead Panel 2, HMMP2MAG 55K, Millipore Corporation, MA, USA).

Supplementary Figures

Figure S1

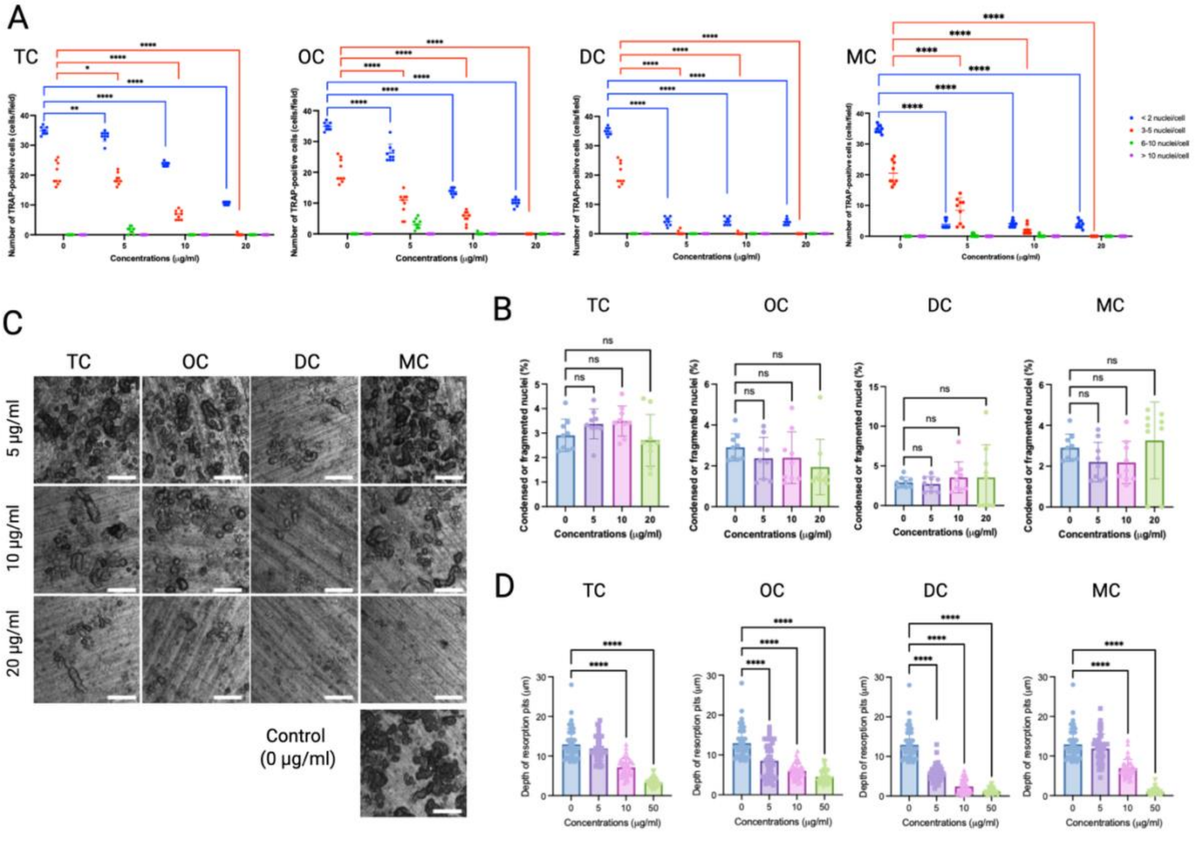

Figure S1. Quantitative analysis of osteoclast morphology and resorption following treatment with TC derivatives. Human osteoclast precursors were cultured in the presence of TC, OC, DC, or MC at the indicated concentrations. **(A)** Quantification of TRAP-positive cells according to the number of nuclei per cell (≤2, 3–5, 6–10, and >10 nuclei). **(B)** Percentage of nuclei exhibiting condensed or fragmented morphology relative to the total number of Hoechst-positive nuclei. **(C)** Representative color laser microscopy images of resorption pits formed on bone slices following treatment with TC derivatives. Scale bars = 100 μm. **(D)** Quantification of resorption pit depth. No significant increase in condensed or fragmented nuclear morphology was detected following TC derivative treatment, whereas all compounds reduced the depth of resorption pits in a concentration-dependent manner. Data are presented as mean ± SD (n = 6). *$P < 0.05$, **$P < 0.01$, ***$P < 0.001$, ****$P < 0.0001$, ns, not significant versus the corresponding 0 μg/mL control.

Figure S2

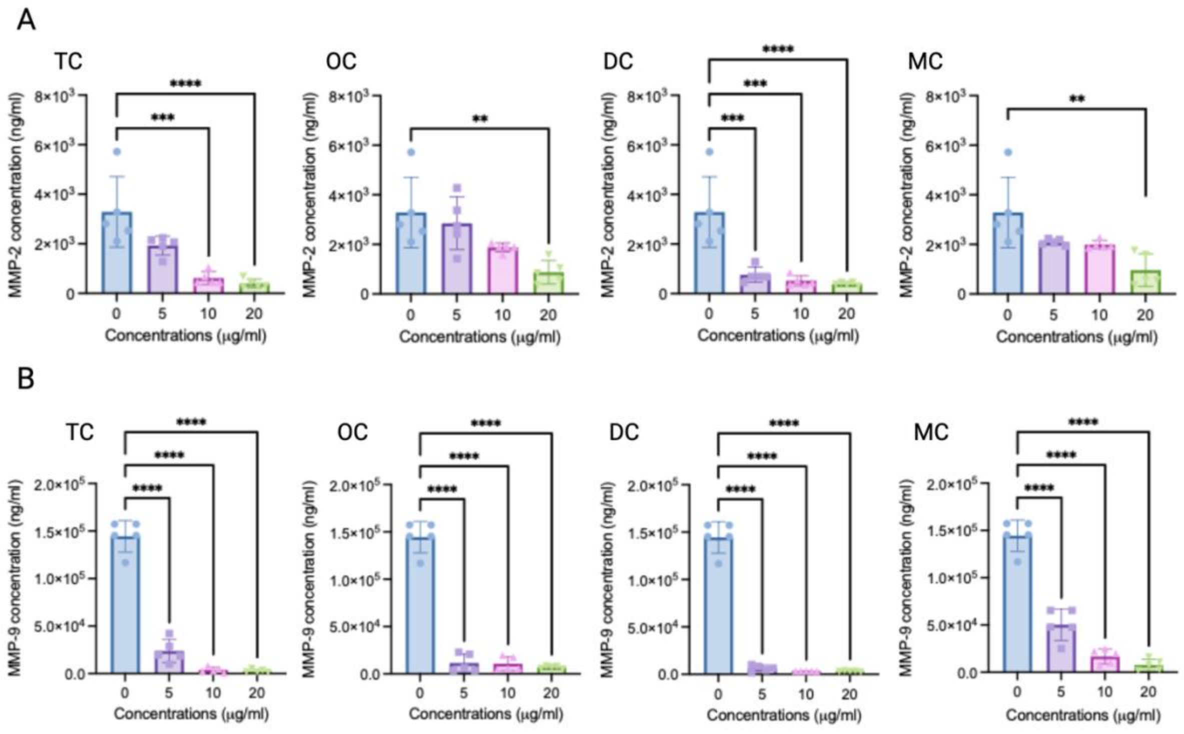

Figure S2. Effects of TC derivatives on MMP-2 and MMP-9 levels in osteoclast cultures. (A) Quantification of MMP-2 levels in cell lysates following 14 days of osteoclast culture on bone slices in the presence of TC, OC, DC, and MC at the indicated concentrations. MMP-2 levels were reduced in cultures treated with higher concentrations of TC (10 and 20 μg/mL), OC (10 and 20 μg/mL), and MC (20 μg/mL), whereas DC reduced MMP-2 levels at all tested concentrations (5, 10, and 20 μg/mL). **(B)** Quantification of MMP-9 levels in cell lysates under the same experimental conditions. All tested TC derivatives reduced MMP-9 levels, with significant effects observed even at low concentrations. Data are presented as mean ± SD (n=5). ** $P < 0.01$, *** $P < 0.001$, **** $P < 0.0001$ compared with the corresponding 0 μg/mL control.

Table S1 Comparison of previously reported bone-related effects of TC derivatives with the findings of the present study.

Compound	Previously reported bone-related effects	Findings in the present study
TC	• High concentrations of TC reduced osteoblast proliferation and metabolic activity [1].<br>• TC promoted osteogenic differentiation-related responses in human MSCs in a comparative study [2].<br>• The affinity of TC for mineralized tissues has been exploited for bone targeting [3].<br>• TC has been reported to inhibit osteoclast formation and resorptive activity in vitro and to affect bone turnover in vivo [4].	Concentration-dependent reduction in osteoclast formation and resorption; reduced ALP-positive area in primary MSC-derived osteoblasts; concentration-dependent effects on MG-63 matrix mineralization; localization to mineralized matrix and developing zebrafish skeleton
OC	• OC has been reported to inhibit osteoclast formation and resorptive activity in vitro and to affect bone turnover in vivo [4].	Concentration-dependent reduction in osteoclast formation and resorption; reduced ALP-positive area; biphasic MG-63 mineralization response; skeletal accumulation in zebrafish
DC	• DC induced osteoclast apoptosis and inhibited osteoclast formation and resorption [5].<br>• DC inhibited MMP-9 activity and RANKL-induced osteoclast differentiation in vitro and in vivo [6].<br>• DC promoted bone repair and altered Wnt signaling in vivo [7].<br>• DC showed osteogenic effects in human MSCs [2].	Most pronounced reduction in osteoclast formation/resorption among the tested derivatives; reduced ALP-positive area; low/moderate concentrations enhanced MG-63 mineralization; skeletal accumulation in zebrafish
MC	• MC inhibited osteoclast differentiation through suppression of MMP-9-mediated histone H3 cleavage [8].<br>• MC showed pronounced osteogenic activity in human MSCs and was associated with Hedgehog signaling [2].	Concentration-dependent reduction in osteoclast formation/resorption; reduced ALP-positive area; biphasic MG-63 mineralization response; skeletal accumulation in zebrafish
Direct comparison across derivatives	Comparative studies have evaluated selected osteogenic responses to multiple TC derivatives, including TC, DC, and MC [2]. However, integrated side-by-side comparisons encompassing osteoclast-related responses, osteoblast-related phenotypes, matrix mineralization, and skeletal accumulation remain limited.	TC, OC, DC, and MC were evaluated side-by-side across osteoclast-, osteoblast-, mineralization-, and skeletal-accumulation models under a unified experimental framework

References

1. Duewelhenke, N.; Krut, O.; Eysel, P. Influence on mitochondria and cytotoxicity of different antibiotics administered in high concentrations on primary human osteoblasts and cell lines. *Antimicrob. Agents Chemother.* **2007**, *51*, 54–63, doi:10.1128/AAC.00729-05.

2. Martin, V.; Bettencourt, A.F.; Santos, C.; Fernandes, M.H.; Gomes, P.S. Unveiling the osteogenic potential of tetracyclines: A comparative study in human mesenchymal stem cells. *Cells* **2023**, *12*, doi:10.3390/cells12182244.

3. Que, Y.; Yang, Y.; Zafar, H.; Wang, D. Tetracycline-grafted mPEG-PLGA micelles for bone-targeting and osteoporotic improvement. *Front. Pharmacol.* **2022**, *13*, 993095, doi:10.3389/fphar.2022.993095.

4. Zhou, X.; Zhang, P.; Zhang, C.; An, B.; Zhu, Z. Tetracyclines inhibit rat osteoclast formation and activity in vitro and affect bone turnover in young rats in vivo. *Calcif. Tissue Int.* **2010**, *86*, 163–171, doi:10.1007/s00223-009-9328-5.

5. Bettany, J.T.; Peet, N.M.; Wolowacz, R.G.; Skerry, T.M.; Grabowski, P.S. Tetracyclines induce apoptosis in osteoclasts. *Bone* **2000**, *27*, 75–80, doi:10.1016/s8756-3282(00)00297-0.

6. Franco, G.C.N.; Kajiya, M.; Nakanishi, T.; Ohta, K.; Rosalen, P.L.; Groppo, F.C.; Ernst, C.W.O.; Boyesen, J.L.; Bartlett, J.D.; Stashenko, P.; Taubman, M.A.; Kawai, T. Inhibition of matrix metalloproteinase-9 activity by doxycycline ameliorates RANK ligand-induced osteoclast differentiation in vitro and in vivo. *Exp. Cell Res.* **2011**, *317*, 1454–1464, doi:10.1016/j.yexcr.2011.03.014.

7. Gomes, K. do N.; Alves, A.P.N.N.; Dutra, P.G.P.; Viana, G.S. de B. Doxycycline induces bone repair and changes in Wnt signalling. *Int. J. Oral Sci.* **2017**, *9*, 158–166, doi:10.1038/ijos.2017.28.

8. Kim, Y.; Kim, J.; Lee, H.; Shin, W.-R.; Lee, S.; Lee, J.; Park, J.-I.; Jhun, B.H.; Kim, Y.-H.; Yi, S.-J.; Kim, K. Tetracycline Analogs Inhibit Osteoclast Differentiation by Suppressing MMP-9-Mediated Histone H3 Cleavage. *Int. J. Mol. Sci.* **2019**, *20*, doi:10.3390/ijms20164038.
